# Rationale and design of the PREDICE project: cost-effectiveness of type 2 diabetes prevention among high-risk Spanish individuals following lifestyle intervention in real-life primary care setting

**DOI:** 10.1186/1471-2458-11-623

**Published:** 2011-08-04

**Authors:** Bernardo Costa, Joan J Cabré, Ramon Sagarra, Oriol Solà-Morales, Francisco Barrio, Josep L Piñol, Xavier Cos, Bonaventura Bolíbar, Conxa Castell, Katarzyna Kissimova-Skarbek, Jaakko Tuomilehto

**Affiliations:** 1 Jordi Gol Primary Care Research Institute, Catalan Health Institute, Primary Health Care Division, Reus-Barcelona, Spain; 2 Catalan Agency for Health Information, Assessment and Quality Generalitat de Catalunya & Health Technology Assessment, SabirMedical S.L., Escola de Doctorat UAB, Barcelona, Spain; 3 Public Health Division. Departament of Health. Generalitat de Catalunya. Barcelona, Spain; 4 Jagiellonian University Medical College. Faculty of Health Related Sciences. Krakow, Poland; 5 Department of Public Health, University of Helsinki & South Ostrobothnia Central Hospital, Seinäjoki, Finland

## Abstract

**Background:**

Type 2 diabetes is an important preventable disease and a growing public health problem. Based on information provided by clinical trials, we know that Type 2 diabetes can be prevented or delayed by lifestyle intervention. In view of translating the findings of diabetes prevention research into real-life it is necessary to carry out community-based evaluations so as to learn about the feasibility and effectiveness of locally designed and implemented programmes. The aim of this project was to assess the effectiveness of an active real-life primary care strategy in high-risk individuals for developing diabetes, and then evaluate its efficiency.

**Methods/Design:**

Cost-Effectiveness analysis of the DE-PLAN (Diabetes in Europe - Prevention using Lifestyle, physical Activity and Nutritional intervention) project when applied to a Mediterranean population in Catalonia (DE-PLAN-CAT). Multicenter, longitudinal cohort assessment (4 years) conducted in 18 primary health-care centres (Catalan Health Institute). Individuals without diabetes aged 45-75 years were screened using the Finnish Diabetes Risk Score - FINDRISC - questionnaire and a 2-h oral glucose tolerance test. All high risk tested individuals were invited to participate in either a usual care intervention (information on diet and cardiovascular health without individualized programme), or the intensive DE-PLAN educational program (individualized or group) periodically reinforced. Oral glucose tolerance test was repeated yearly to determine diabetes incidence. Besides measuring the accumulated incidence of diabetes, information was collected on economic impact of the interventions in both cohorts (using direct and indirect cost questionnaires) and information on utility measures (Quality Adjusted Life Years). A cost-utility and a cost-effectiveness analysis will be performed and data will be modelled to predict long-term cost-effectiveness.

**Discussion:**

The project was intended to evidence that a substantial reduction in Type 2 diabetes incidence can be obtained at a reasonable cost-effectiveness ratio in real-life primary health care setting by an intensive lifestyle intervention. As far as we know, the DE-PLAN-CAT/PREDICE project represents the first assessment of long-term effectiveness and cost-effectiveness of a public healthcare strategy to prevent diabetes within a European primary care setting.

## 1. Background

The growing impact of Type 2 Diabetes (T2D) in high income countries requires the introduction of better and more secure treatments, but also pushes towards the development of new preventing strategies to reduce the incidence and prevalence of the disease [1,2]. Many studies have been published on the efficacy of new treatments of diabetes or its complications but there is still scarce information on its prevention. As a general rule, prevention strategies are more efficient than treating, though there is a great reluctance from a policy perspective to transfer funds from one strategy to the other [3]. Diabetes prevention has not been prioritized and is still not high in the health policy agenda, despite evidence showing its ability to prevent new cases and even to prevent the metabolic syndrome [4-6]; the consequences of not including these polices are costly on the health side, but also on other aspects of society, given the long term inability consequences of diabetes, not to say of its skyrocketing incidence.

A metanalysis published in 2007 underlined the effectiveness of several pharmacologic and non-pharmacologic (lifestyle) interventions to prevent or at least delay the incidence of T2D in patients with impaired glucose tolerance [7]. More recently, a systematic review showed that life-style changing interventions could be the most cost-effective [3], though; most of the evidence came from modelling techniques (used disease progression model to represent the long term health benefits and costs) and not from effectiveness evaluation programs. The economic evaluation of the Finnish Diabetes Prevention Program, where the event rates (both cardiovascular and cerebrovascular) were taken from the UKPDS study, showed that the intervention was cost-saving from the healthcare system payers' viewpoint and cost effective from societal perspective [8]. Even if based on modelling, important data were presented there on the costs of the interventions, which are of interest for our study.

In The Netherlands, a country devoting a large share of Gross Domestic Product to preventive programs, costs of two lifestyle modifying programs (intensive versus community counselling on obese individuals) were evaluated: 7 to 30 individuals were needed to prevent a new case of diabetes in the intensive cohort, but from 30 to 1500 were needed to reach the same effectiveness ratio at the community level. Though the costs were grater in the intensive group (5.000 to 21.000€) when compared to the community (2.000 to 9.000€), the cost effectiveness ratio was much more favourable in the intensive group (3.100 to 3.900€/QALY versus 3.900 to 5.500€/QALY) [9].

It is accepted that a well designed and implemented program on T2D prevention is more effective (and even cost-effective) than doing nothing [10,11]. However, as suggested by the US Diabetes Prevention Program (DPP) the cost depends greatly on the program implementation costs and the contextual characteristics (health care system characteristics) [12,13]. In fact, two different reviews of the same program did not reach the same conclusions on the cost-effectiveness of using metformin, and despite it could be argued that the DPP had a too expensive life-style intervention strategy, it is evident that the efficacy and efficiency in this programs may vary depending on the wealth of the participants but also on their dietary habits and the ability of an intervention to significantly reduce weight [14].

In all, most of the economic evaluations consider medicines and modelling techniques instead of collecting real data on effectiveness and resources used during the intervention. In addition primary prevention is only marginally assessed. Despite it may be difficult to prove, it seems plausible to prove that prevention is efficacious, effective and efficient. The obvious limiting factor is the capacity to prove short and long term benefits, and the ability to reduce uncertainty on the effect of a long-lasting intervention [15]. In such context, it is crucial to define both the target population and the resources availability (time, capital and labour).

## 2. Design and methods

### Hypothesis

Preventive interventions are both effective and efficient in high risk individuals to develop T2D.

### Objectives

1. The primary objective is to prove the cost-effectiveness of the DE-PLAN-CAT life-stile intervention, where a usual-care informative strategy was compared with an intensive intervention to prevent T2D in high risk individuals.

2. Secondary objectives were to assess the quality of life of such individuals with the validated 15D questionnaire before, during and after the intervention.

3. Third, to analyse the cost-effectiveness of such intervention on a generalized level, by using simulation models.

### Study Design

Using data from the DE-PLAN-CAT study, different sub-analysis will be performed:

1. Direct and indirect cost analysis of both strategies

2. Cost-effectiveness analysis of both strategies

3. Cost utility analysis of both interventions

4. Long term modelling simulation on the cost-effectiveness of both interventions.

### Setting

**Figure 1:**
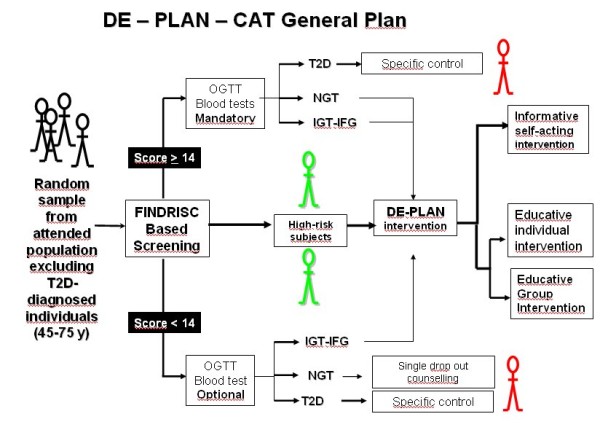
**General plan of DE-PLAN-CAT project**.

The original DE-PLAN-CAT study was performed in 18 Spanish primary health-care centres in Catalonia (Catalan Health Institute) (figure 1).

### Sample size calculation

The participating centres were selected in a stratified manner covering primary care services of 315,703 inhabitants (4.5% of the population in Catalonia). The necessary sample of participating subjects was obtained mainly from the computerized list of the public primary health care system. In addition, several participating teams noticed the initiative and also contacted with local authorities or pharmacies in view of implement the study. The sample size was calculated regarding available data on diabetes incidence in high-risk Catalan population [16]. Therefore, it was assumed that the mean annual incidence in the usual care intervention group would be 7.5% and the mean expected incidence in the group of maximum impact (intensive intervention group) would be 3.25% (50% reduction in the yearly rate). Basic estimates for calculating the sample size also included the following hypothesis: 35% positive high-risk screenees by the FINDRISC, 10% negative screenees with prediabetes diagnostic criteria at blood test, 20% of individuals having undiagnosed diabetes and a theoretical distribution between cases in the usual care, intensive individualized and intensive group interventions close to 1:1:1. Thus, allowing for a discontinuation rate of 30% it was expected to screen a minimum of 1650 subjects and include a minimum of 550 in the intervention with a type 1 error of 5%, with 80% power (beta = 20%) at the two-tailed 5% significance level.

### Subjects and Clinical Intervention

According to the original study, an active public health programme was applied to a Mediterranean population based in Catalonia (Spain). Caucasian individuals (n = 2054) without known diabetes aged 45-75 years were evaluated by general practitioners in 18 primary healthcare centres. The FINDRISC questionnaire was used for screening as it has been well validated across Europe to elicit the risk of developing T2D. This 8-item questionnaire (0-26 points in the used version), collects information on age, sex, weight and height, waist circumference, use of concomitant medication (blood pressure), history of blood glucose disorders, physical activity and daily consumption of vegetables, fruits or berries.

All subjects showing to have a high risk of T2D (FINDRISC score > 14 and/or non-diabetic hyperglycaemia at blood test) were included in the lifestyle intervention study (n = 552, 4-year follow-up). All individuals with severe psychiatric disease, chronic kidney or liver disease or blood disorders, were excluded.

Anthropometric parameters were determined by trained nurses. Body weight and height were measured in light clothing and without shoes. Waist circumference was measured midway between the lowest rib and the iliac crest. A 2-h 75-g oral glucose tolerance test was carried out according to the World Health Organization (WHO) standards with measurements of fasting plasma glucose and 2-h post-load glucose. Simultaneously lipid profile and HbA1C determinations were performed. These measurements were repeated at the annual follow-up visits.

**Figure 2:**
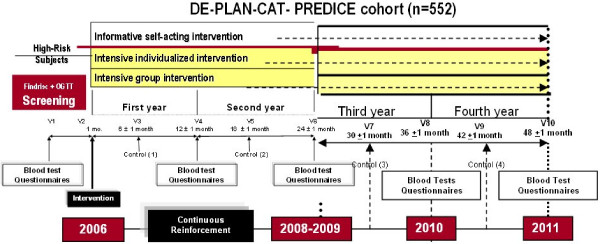
**Diagram of the DE-PLAN-CAT/PREDICE cohort**.

The intervention consisted of two steps (initial and further reinforcement) and two elective interventions (informative or intensive). In the usual care informative intervention, each participant was told about the risk of developing diabetes and its possible health consequences, and a standard training material was provided; at their next visits to the physician, standard reminders were given. The intensive intervention consisted of a six-hour educational program (divided in 2 to 4 sessions) to be performed either individually or in small groups (5 to 15 people). The intervention was based on motivation, peer support and positive feed-back and the core of the information given was about the risks of having diabetes, the Mediterranean diet together with nutritional advice, the beneficial effects of exercise and tobacco cessation advice. Participants were reminded (telephone call) about the date and hour of their next group session to ensure compliance, and every 6-week (minimum) they were contacted (also by telephone). In 2 out of 8 centres SMS sending was preferred for continuous intervention. Process-based evaluation of the individual risk and response to the intervention were provided to encourage the lifestyle modification. Figure 2 describe the study design.

### Economic Analysis

Specific DE-PLAN approach for cost assessment was designed incorporating WHO-CHOICE concept for analyzing and presenting resources used at different levels and in two periods of programme implementation. These are:

a) Start-up and post start-up costs

The start-up period covers pre-implementation phase of the programme including: planning, consensus meetings, organizing, training of the personnel, printing materials, informing. These costs are spent only once for the organization of the prevention programme in each country. Programme can be extended to another region without necessity to cover again the total amount of start-up costs.

The post start-up period covers implementation and running the programme. These costs are analyzed to mirror the main steps of DE-PLAN project:

Step 1: Selection of the subjects

Step 2: Intensive intervention

Step 3: Continuous intervention

b) Cost of "management level" and "participant level"

Cost at management level consider costs for coordinating of the intervention in each participating centre including planning, organizing, monitoring and supervision of the intervention, training of the personnel during both start-up and post start-up periods of the programme.

Costs at participant level include all resources used at the point of delivery of the intervention (provider of the intervention) such as: costs of screening, testing with the oral glucose tolerance test and other biochemical examinations, educating, exercising etc. Changes in use of health services due to intervention are also considered.

Within the presented structure of costs assessment the following 8 forms were used in order to collect data to assess direct (medical and non-medical) and indirect costs of the intervention.

1. E01 Form: It evaluates the resources used in the screening which includes the identification and selection of the participants in the intervention programme. The programme measures the time needed to complete the FINDRISC questionnaire, the cost in human resources to carry out the activity and other specifications such as the time employed for the analytical tests. It also measures the time to complete the intensive intervention on lifestyle identifying all the professionals involved; telephone calls and material provided (leaflets, photocopies, etc.). A randomised sample from each centre will be evaluated.

2. E02 Form: It also records the number of contacts with the health system that each participant required to finish the stage, the cost of materials used for blood tests (personnel not included), the number of oral glucose tolerance test carried out, telephone calls and average duration of the calls, writing material provided and photocopies, extra-time and number of personnel and dates of the specific training in the programme of the personnel collaborating in the study.

3. E03 Form: The purpose is to assess the change in (i) costs of medical care outside the DE-PLAN programme, which the intervention might cause- by either side effects or by improvement of health and (ii) indirect costs to society. This form adds up the use of different types of health services by DE-PLAN participants during the programme. In other words information collected with this form is on the number and duration of hospitalizations, ordinary visits to health centres, the number of visits to emergency units, to specialists and other healthcare providers, whether the person is currently working, if they have been on sick leave and for how long. The period of data considered is for the year before the participant entered the study and for the period of programme implementation.

4. E04 Form: The purpose of the form supplied to the different centres participating in the program is to identify an average (per group and per participant) personnel time necessary to perform the intensive intervention. This form collects data on the intensive intervention, noting the duration and characteristics of the intervention group sessions as well as the personnel who gave these sessions, the topic and average length of the session.

5. E05 Form: This form deals with the continuous intervention questionnaire which collects the average number of participants, total number of follow-up phone calls and the time expended by professionals.

6. E06 Form: The purpose is to collect data on management of the intervention in participating centres (including resources necessary for planning, organising, monitoring, supervision and training of personnel performing the interventyion). It collects data regarding personnel differentiating among qualifications, responsibilities, effective time spent dedicated to the project the total length of time in which they are committed to the project. It includes also materials and supplies at market price; transport costs (travel expenses, parking, motorway tolls, etc). The costs of the telephone and other means of messaging (e.g. SMS) will be estimated. It also includes estimates of the cost of the carrying out of the screen tests (exclusively).

7. E07 Form: This refers to the macroeconomic information about the Spanish and Catalan health systems (for European Project purposes).

8. E08 Form: This collects data necessary to identify costs incurred by participant due to intervention including: the cost of transport to the centre or the time spent travelling; the time spent going to gymnasiums or swimming pools as part of the programme; and costs to the participants which do not fall within the programme (books, gym or sports centre fees, equipment, classes and so on). Also included are the changes in the monthly cost of food, drink and restaurant bills before and after the educational intervention.

### Organization, Data Collection and Analysis

#### Organization

A multidisciplinary coordinating committee was established (DE-PLAN-Catalonia Coordinating Committee) to implement and coordinate the study, At the Catalan Institute of Health, every providing centre is composed of many independent 'teams' (physician, nurses and other staff that manage their own activity though coordinated at a centre level); at each participating centre one team was invited to join the committee together with the managing and specialized professionals). Data were collected and monitored electronically. All centres were computerized regarding data collection and monitoring. The Research Unit of Reus-Tarragona provides, besides coordination and steering, methodological support and treatment of the data.

### Data Collection

Data were initially collected on paper forms by the collaborating investigators, and an independent non-epidemiologist introduced the data into the database. To ensure quality and avoid discrepancies, files were reviewed by an expert epidemiologist. In case of data inconsistencies, the teams were required to solve the problem by a "query - response" electronic form. An additional check was performed after entering the data in the database, to avoid inconsistencies due to any mistakes in the data input.

### Analysis

Cost-effectiveness (and cost-utility) ratios will be analyzed for both interventions (informative vs. intensive), and an incremental cost-effectiveness (cost-utility) ratio will also be calculated. Direct and indirect costs will be accounted for as described in the above mentioned forms and measured according to reliable available rates in the Catalan health service, thus providing internal and external consistency.

The main outcome measures will then be (for both arms and the difference): average costs, average effectiveness, average cost-effectiveness and cost-utility ratios, and incremental cost-effectiveness and cost-utility ratios. The comparator for intensive and informative study group is control group (no intervention). Utility measures will be extracted from the 15D questionnaire, and from there we will elicit QALYs gained for both interventions [17,18].

For the long term simulation model, we will use input from the trial and run the model to predict long term costs, effects and incremental cost-effectiveness ratios, using both MS Excel files and *TreeAge Data *[19].

## 3. Discussion and points of interest

This study will present a new analysis of the effectiveness of T2D prevention. If data confirm our hypothesis, this will help policymakers' to allocate larger investments in prevention rather than treatment. It is important to note that data feeding the analysis will be collected from a real life environment, and despite the trial will be set up on a research basis and under a quasi-controlled environment, we believe we can assume these will be effectiveness data. In fact the efficiency analysis will be done including all interventions aimed to be sustained in the large-scale implementation phase.

Of note also, the study includes non-service cost data: not only has this eagerly been attempted before, but also it will provide important and relevant information for policy-making.

Finally, we would like to stress that we aim to publish the results of this and accompanying studies to inform policy making and to induce a needed change.

## Competing interests

The authors declare that they have no competing interests.

## Authors' contributions

BCP wrote the research proposal which was approved by the *Instituto de Salud Carlos III *for funding and the manuscript winner of the first prize for innovation and organization in primary health care processes. JCV also wrote the application funded by the CAMFiC. The economic analysis was written by OSM and KKS. RSA received a predoctoral fellowship from the Jordi Gol Primary Care Research Institute (Catalan Health Institute). All authors read and approved the final document of this protocol.

## Pre-publication history

The pre-publication history for this paper can be accessed here:

http://www.biomedcentral.com/1471-2458/11/623/prepub

## Aknowledgements

### Institutional approval and support of a public nature

This project was funded by the Commission of the European Communities, Directorate C--Public Health (grant agreement no. 2004310) and also by the Health Institute Carlos III, Spanish Ministry of Health, grant agreements FIS (PI05-033/PS09-001112), the Department of Health, Generalitat de Catalunya and a research grant from the Polish Ministry of Science and Higher Education (financial sources for research in years 2008-2009). The research ethics board of the Jordi Gol Research Institute (Barcelona, Spain) approved the protocol and all participants gave written informed consent. In 2010 The PREDICE project was awarded the first prize for "Innovation and organization in primary health care processes" by the Catalan societies of General Practitioners (CAMFiC), nursing (AIFICC) and the Department of Health (Generalitat de Catalunya).

### Members of the DE-PLAN-Catalonia Coordinating Committee

Bernardo Costa, Bonaventura Bolíbar, Conxa Castell, Francisco Barrio, Jerónimo Jurado, Jordi Salas, Josep Lluís Piñol, Mònica Bulló, Joan Josep Cabré, Teresa María Llauradó, Xavier Cos, José Antonio Fernández, Lara González, Xavier Mundet, Claustre Solé, Teresa Mur, Agueda Castaño, Sònia Sarret, Montserrat Torres, Marifé Àlvarez, Montserrat Cot, Inmaculada Ojeda, Jacint-Àngel Caula, Montserrat Ortigas, Marta Roura, Maria Pastoret, Francesc Pujol, José Luis Bueno and Ramon Sagarra.

### Participating reference centres

Reus-Tarragona, Barcelona (Carmel, Sant Martí, Santa Eulàlia, Terrassa-Sud), Lleida and Girona (Blanes, Figueres-Olot).
